# Leveraging implementation science theories to develop and expand the use of a penicillin allergy de-labeling intervention

**DOI:** 10.1186/s12913-024-11364-7

**Published:** 2024-08-26

**Authors:** Esra Alagoz, Megan Saucke, Prakash Balasubramanian, Tyler Liebenstein, Sujani Kakumanu

**Affiliations:** 1 Department of Surgery, University of Wisconsin-Madison, 600 Highland Avenue, Madison, 53792-7375 WI USA; 2 William S. Middleton Veterans Memorial Hospital Madison, Madison, WI USA; 3 Department of Medicine, Division of Allergy, Pulmonary and Critical Care, University of Wisconsin-Madison, Madison, WI USA

**Keywords:** Penicillin, Drug allergy, Implementation science, Qualitative data, Mixed-methods, Multi-methods, Quality improvement, Healthcare quality

## Abstract

**Background:**

Penicillin allergy is the most frequently reported drug allergy, yet most patients can tolerate the drug if challenged. Despite this discrepancy, large scale penicillin allergy de-labeling interventions have not been widely implemented in many health care systems. The application of a multi-method implementation science approach can provide key tools to study this evidence to practice gap and provide insight to successfully operationalize penicillin allergy evaluation in real-world clinical settings.

**Methods:**

We followed a four-step process that leverages qualitative analysis to design evidence-based, actionable strategies to develop an intervention. First, we specified the clinician-perceived barriers to penicillin allergy de-labeling (intervention targets). We then mapped intervention targets onto Theoretical Domains Framework (domains and constructs) and found the root causes of behavior. Next, we linked root causes of behavior with intervention functions (BCW). In the final step, we synthesized participants’ suggestions for process improvement with implementation strategies aligning with the intervention functions.

**Results:**

Evidence-based strategies such as focused education and training in penicillin allergy evaluation can address knowledge and confidence barriers reported by frontline clinicians. Other key strategies involve developing a system of champions, improving communications systems, and restructuring the healthcare team. Implementation mapping can provide a powerful multi-method framework to study, design, and customize intervention strategies. Conclusion: Empowering clinicians beyond allergy specialists to conduct penicillin allergy assessments requires designing new workflows and systems and providing additional knowledge to those clinicians.

**Supplementary Information:**

The online version contains supplementary material available at 10.1186/s12913-024-11364-7.

## Introduction

The detrimental impact of the penicillin allergy label on patient care, health care utilization, and antimicrobial prescribing practices has been well described [1–3]. Despite its prevalence, studies have shown that up to 90% of patients with the label of penicillin allergy can tolerate a penicillin antibiotic if challenged [4]. Interventions that offer systemic evaluation of patients with a penicillin allergy diagnosis have been developed to address this evidence to practice gap of care [5]. These tools typically include the following: (1) identifying penicillin allergic patients (2) using risk factors such as time and symptoms of past reactions to risk stratify patients regarding the future risk of a reaction to penicillin (3) offering an oral challenge to penicillin, preceded by skin testing when appropriate. Several studies have reported effective algorithms, scoring systems, and challenge protocols that can be implemented at point of demand, including use of telehealth and/or e-consult mechanisms [6–9]. Although these innovations exist, further work is needed to address the barriers and contextual factors that may affect key implementation outcomes such as scalability, sustainability, feasibility, and efficacy of large-scale interventions. In addition, variations in clinical settings and patient populations will impact how penicillin allergy evaluations are conducted. To further close the evidence to practice gap, implementation science theories can be applied to study both the clinical efficacy of the intervention and key implementation outcomes related to reach, sustainability, feasibility, and fidelity of a penicillin allergy de-labeling intervention.

Developing theory-based interventions that support healthcare professionals in modifying their clinical practices according to evidence-based recommendations is an important strategy in implementation science. Although the need for evidence-based interventions is well recognized, only a few studies employ social and behavioral theories when developing or implementing interventions [10–12]. Building on our prior work for which we interviewed inpatient and outpatient clinicians and described the key barriers to the implementation of our penicillin allergy evaluation initiative and Clinical Decision Support Tool (CDST) [13] (See supplementary files for the interview questions), we have leveraged a multi-methods approach to determine best practices to improve implementation of penicillin allergy de-labeling. This evidence-based process called implementation mapping (IM) translates our qualitative, contextual data to specific strategies to design an effective intervention, specifically for penicillin allergy de-labeling.

## Methods

**Fig. 1:**
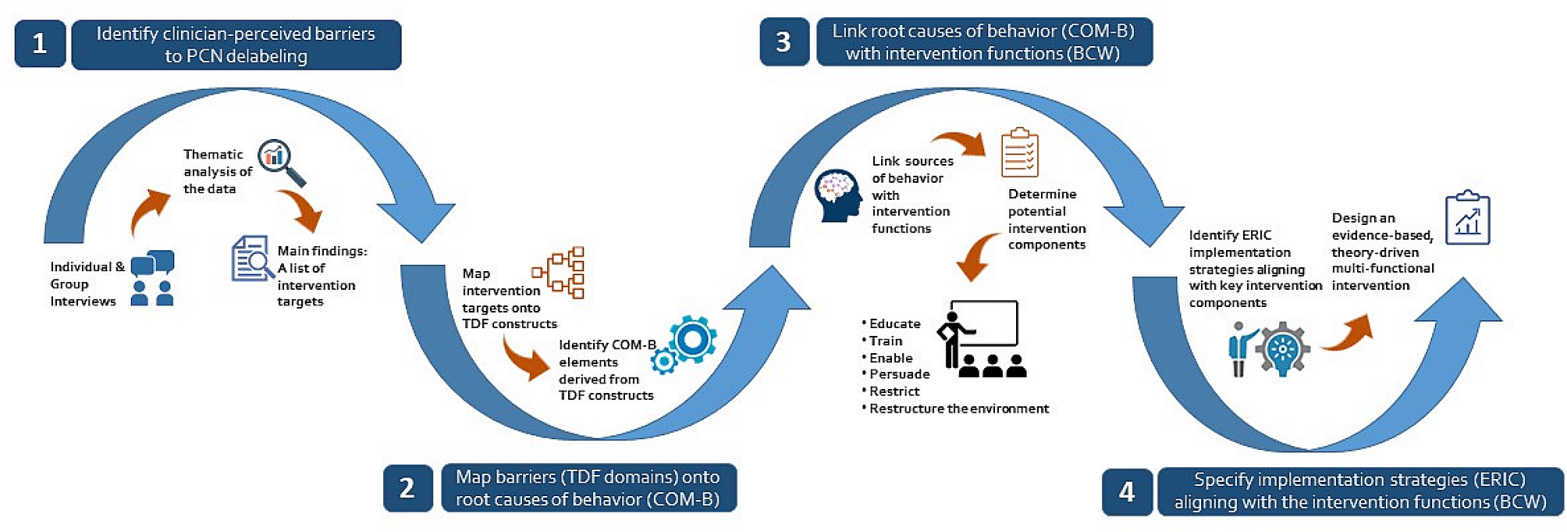
Four step process for implementation mapping. This describes the process for mapping qualitative data (identified barriers) to design evidence-based actionable strategies to improve the application of penicillin allergy evaluation in real world settings. TDF: Theoretical Domains Framework; COM-B: Capability, Opportunity, Motivation- Behavior; BCW: Behaviour Change Wheel; ERIC: Expert Recommendations for Implementing Change

Most studies that build on theories to develop behavioral change interventions employ a step-by-step approach during the design process. For example, French et al. [10] utilized a 4-step approach to develop a cohesive behavioral intervention, Hrisos et al. [11] employed a 6-step process to design two theory-based interventions, and Foy and colleagues [12] described a 10-step iterative process to develop their intervention. We developed our IM process with the following steps: first, specify the target behavior; second, select an appropriate theoretical framework; and third, map the target behaviors onto behavior change techniques and fourth, choose the appropriate methods of delivery of techniques (Fig. 1).

Our goal was to design a multi-modal intervention that targets clinicians’ behavior and incorporates de-labeling practices into their clinical workflows. Our process (Fig. 1) can be outlined by the four steps below:

### Step 1. Specify the clinician-perceived barriers to penicillin de-labeling (intervention targets)

In an earlier stage of our research, we conducted individual and group interviews with 20 clinicians from multidisciplinary inpatient and outpatient healthcare teams within a single site veteran’s hospital. Our goal was to explore workflows and contextual factors influencing identification and evaluation of patients with penicillin allergy. A more detailed description of the qualitative methodology has previously been published [10]. We coded the data using thematic analysis [14] to identify the major barriers to a risk-based penicillin de-labeling protocol in inpatient and outpatient settings.

### Step 2. Map intervention targets onto TDF theory (domains and constructs) and find the root causes of behavior (COM-B)

We then mapped these intervention targets onto domains and constructs within the Theoretical Domains Framework (TDF) [15] to organize the major barriers to implementing the de-labeling protocol (Table 1). This second step elucidated the hospital context and culture, interdependent nature of workflows, and the individual clinicians’ perceptions and behaviors that hinder their engagement with de-labeling and further identified targets for change. These findings highlighted the need to employ a systemic approach that addresses each of the domains influencing clinician behaviors regarding penicillin allergy de-labeling. Each barrier to target behavior categorized under a TDF domain was then mapped onto the sources of the behavior in the Behaviour Change Wheel (BCW) [16]. Theory of BCW posits that behaviors are a function of three underlying factors: *Capability* (C) relates to individuals’ psychological and physical capacity to perform behaviors, including having necessary knowledge and skills. *Opportunity* (O) is linked to external factors that enable or prompt behaviors such as environmental and cultural context. *Motivation* (M) is connected to internal processes such as habits, impulses, emotions, and logical reasoning that shape decision-making and behavior. Two types of motivation are described in BCW. *Reflective* motivation relates to the high cognitive processes, such as beliefs, values and goals and can be addressed by increasing knowledge. On the other hand, *automatic* motivation involves processes that are linked to emotional responses, habits, impulses and inhibitions which could be addressed through habit formation.

**Table 1:** First three steps of implementation mapping. TDF: theoretical domains Framework; COM: capability, opportunity, motivation; BCW: Behaviour Change Wheel; PCN: penicillin; CDST: clinical decision support tool; EHR: electronic medical record; F2F: face to face

Step 1	Step 2			Step 3
Barrier/ Intervention targets (interview data)	Intervention Domain (TDF)	Intervention construct (TDF)	Target root cause (COM)	Intervention functions (BCW)
• Low knowledge of safety and benefits of pcn allergy de-labeling	Knowledge	Scientific rationale	Capability/ psychological	• Education • Training • Enablement • Persuasion
• Varying awareness of the clinical support tool for de-labeling (CDST) and location in EHR.		Procedural knowledge	Capability/ psychological	• Education • Training • Enablement
• Lack of training and frequent practice with risk stratification, drug challenges, and treating adverse reactions	Skills	Skills development	Capability/ psychological	• Education • Training • Enablement
• Low comfort level to de-label and challenge patients without approval from Allergy	Beliefs about Capabilities	Perceived competence	Motivation/ reflective	• Education • Persuasion
• Fear of poor outcomes such as allergic reaction, delayed discharge, and/or professional censure	Beliefs about Consequences	Outcome expectancies	Motivation/ reflective	• Education • Persuasion
• Pressure to discharge patients quickly • Time constraints and competing tasks	Environmental Context and Resources	Environmental stressors	Opportunity/ physical	• Environmental restructuring • Enablement
• Lack of staffing to de-label patients with pcn allergy • EHR structure impedes finding accurate allergy history and using CDST		Resources / material resources	Opportunity/ physical	• Environmental restructuring • Enablement
• No clear expectation to de-label patients. • Misperception of efficiency to use alternative antibiotics • Lack of multi-disciplinary inpatient rounding and F2F communication • Non-F2F communication perceived as non-urgent leading to delays in tasks related to drug challenges		Organizational culture /climate	Opportunity/ physical	• Environmental restructuring • Restriction • Enablement
• Unspecified roles around ownership of the process, leading to delays in process initiation and completion	Professional Role and Identity	Professional Role	Motivation/ reflective and automatic	• Education • Persuasion • Environmental restructuring • Enablement

### Step 3. Link root causes of behavior (COM-B) with intervention functions (BCW)

Once we assigned a root cause (COM) to each barrier, we looked at the intervention functions associated with each factor (Table 1). Behavior change theory defines intervention functions as broad categories of planned activities one can do to change a behavior. Each intervention function could influence one or more of the underlying factors. For example, behaviors relating to capability can be intervened on through education, training, and enablement, while behaviors shaped by opportunity can be intervened on through environmental restructuring, enablement, and restriction. In this paper we’ve only reported intervention functions reflected in our interview data. For the full list and definitions, please see Michie et al. [16].

### Step 4. Specify ERIC implementation strategies aligning with the intervention functions

**Fig. 2:**
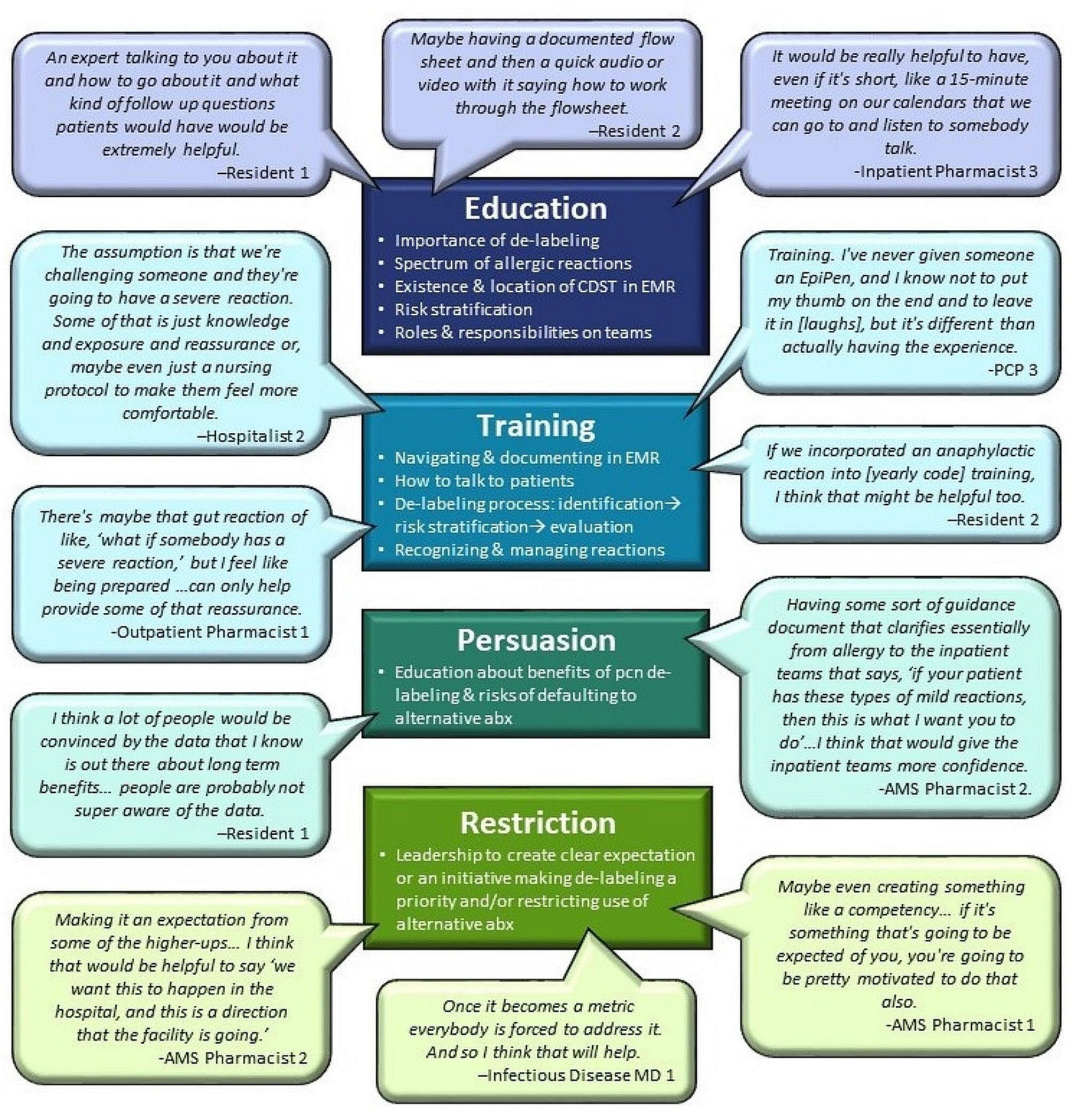
Participant quotes organized by intervention functions (education,* training*,* persuasion*,* restriction)*. CDST: Clinical Decision Support Tool, EMR: Electronic Medical Record, Pcn: Penicillin, Abx: Antibiotics

**Fig. 3:**
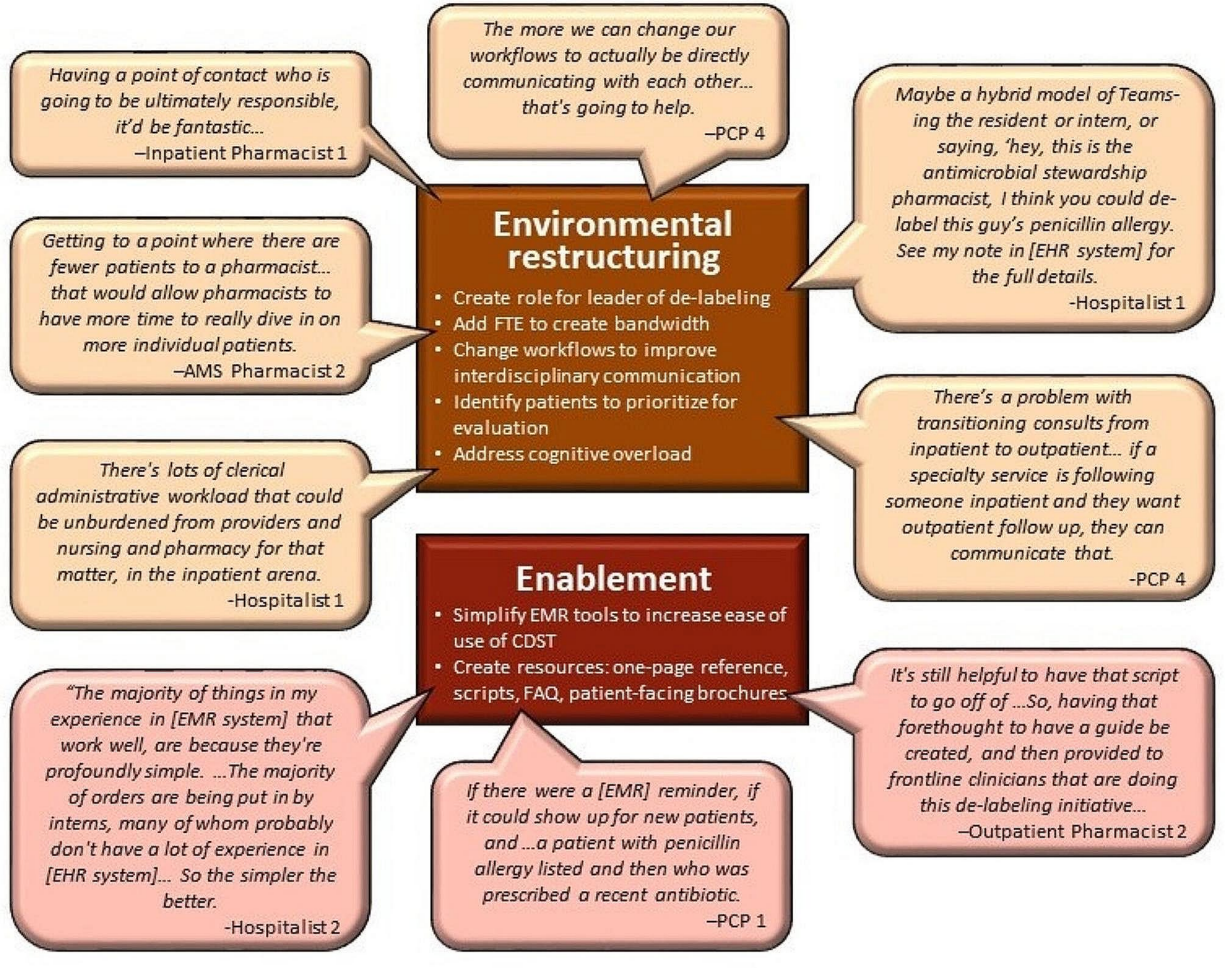
Participant quotes organized by intervention functions (environmental restructuring,* enablement).* CDST: Clinical Decision Support Tool, EMR: Electronic Medical Record, Pcn: Penicillin, Abx: Antibiotics

Next, we returned to the interview data we previously gathered from multidisciplinary inpatient and outpatient healthcare teams. We synthesized the data coded for ‘process improvement.’ This coded data included participants’ suggestions to improve the process of implementing penicillin de-labeling into the workflow. As the next step, we mapped these findings onto relevant intervention functions in the BCW and used interviewees’ suggestions as a starting point to ensure that our intervention responds to the needs of key stakeholders (see Figs. 2 and 3).

Separately, we brainstormed interventions related to each intervention function and cross-referenced them with our qualitative findings. At this point, we also referred to ERIC Implementation strategies [17] consisting of a compilation of 73 implementation strategies verified by the experts in the field of implementation science. This process enabled us to develop intervention ideas informed by stakeholder input, behavioral change theories, and evidence-based implementation strategies (Table 2).

**Table 2:** Fourth step of implementation mapping and components of the intervention. CDST: clinical decision support tool, EHR: electronic medical record, PCN: penicillin, AMS: antimicrobial stewardship

Intervention function (BCW) (definition)	Barrier/intervention target	Step 4	
		ERIC implementation strategy	Key intervention component
Education *(increasing knowledge or understanding)*	• Low knowledge of safety and benefits of pcn allergy de-labeling • Varying awareness of CDST existence and location in EHR	• Conduct educational meetings and educational outreach visits • Develop and distribute educational materials • Intervene with patients to enhance uptake	Develop and distribute: • Patient education: Brochure on the benefits of pcn allergy de-labeling • Health professional education: One pager how-to on the process of allergy evaluation and FAQ • Session 1 of small group training: increasing scientific knowledge of pcn allergy de-labeling
Training *(imparting skills)*	• Lack of training and frequent practice with risk stratification, drug challenges, and treating adverse reactions • Low comfort level to de-label and challenge patients without approval from Allergy • Fear of adverse outcomes such as allergic reaction or delayed hospital discharge	• Use train-the-trainer strategies • Conduct ongoing training • Make training dynamic • Provide clinical supervision	• Session 2&3 of small group training: develop individual skills (physicians and pharmacists) in using CDST and determining risk level for de-labeling • Develop and practice a “script” for use in counseling or explaining pcn de-labeling procedure to patients • Develop a reporting system for patients who “fail” challenges to allow for education on complex patients • Create asynchronous simulation for treating reactions in the ambulatory and inpatient setting
Modelling *(providing an example for people to aspire to or imitate)*	NA	• Shadow other experts​ • Capture and share local knowledge​ • Identify and prepare champions​ • Model and simulate change	• Designate a PCN Allergy Taskforce to help with modeling of pharmacists • Identify champions within the AMS team to provide “at the elbow” Q&A support • Designate a hospitalist team to pilot and champion workflow for pcn allergy de-labeling upon admission
Persuasion *(using communication to induce positive or negative feelings or stimulate action)*	• No clear expectation to de-label patients.	• Develop and implement tools for quality monitoring/Develop and organize quality monitoring systems • Audit and provide feedback • Conduct local consensus discussions • Build a coalition • Inform local opinion leaders	• Develop a data tracking tool to collect clinical outcomes of patients with pcn allergy and present results at regular training sessions
Restriction (*Using rules to reduce the opportunity to engage in competing behaviors*)	• Easier to use alternative antibiotics	• Mandate change	• Provide an aggregate reporting workbench through the AMS committee on how many patients were de-labeled. • Work with national committees to develop feasible metrics to incentivize change
Environmental restructuring *(changing the physical or social context)*	• Unclear roles around ownership of the process, leading to delays in process initiation and completion • Lack of staffing to de-label patients with pcn allergy • Pressure to discharge patients quickly • Time constraints and competing tasks • Lack of multi-disciplinary inpatient rounding and F2F communication • Non-F2F communication is seen as non-urgent and leads to delays in tasks related to challenges	• Revise professional roles • Create new clinical teams • Facilitate relay of clinical data to providers • Provide ongoing consultation	• Provide increased scope to AMS pharmacists to triage and help complete these consults • Create competencies for inpatient pharmacy and AMS pharmacy so that roles and responsibilities are clearly evaluated. • Address staffing demands with inpatient and outpatient supervisors to allow for protected time to devote to pcn allergy de-labeling • Provide a “pop-off valve” with specified capacity limitations for times of high hospital census (triage low-priority patients to outpatient evaluation) • Increase use of e-consults so that any recommendations needed from allergy can be made earlier in the hospital stay
Enablement *(increasing means/reducing barriers to increase capability or opportunity)*	• EHR structure impedes finding accurate allergy history and using CDST	• Assess for readiness and identify barriers and facilitators • Tailor strategies • Change record systems • Remind clinicians	• Develop specific note template and order set to prompt users to obtain key components of the history • Work with EHR vendors to develop designations for removal of pcn allergy within the problem list or allergy field and scalable decision support tools • Work with the hospital informatics teams to operationalize and standardize de-labeling order sets amongst emergency, inpatient and ambulatory menus

### Ethical considerations

UW-Madison Institutional Review Board approved the study and granted minimal risk status. Participants were provided with written information about the study, told participation was voluntary, and given the opportunity to ask questions. Identifying information was removed from transcripts to ensure confidentiality. All participants provided written consent for participation.

## Results

Our results demonstrated a number of intervention targets that could be addressed by six intervention functions: education, training, persuasion, restriction, environmental restructuring, and enablement. The participant quotes mapped on to each intervention function can be found in Figs. 2 and 3.

### Education

All participants talked about the need to disseminate information and increase knowledge for any clinical team members that would be involved in the evaluation process. This education should include information about the spectrum of allergic reactions and how the risk level of evaluation is not any higher than other routine patient care procedures. For the specifics of how to evaluate patients with penicillin allergy, participants discussed the need for a demonstration about the existence of the clinical decision support tool (CDST) and where it can be accessed. Participants also thought it was important for education to include information about the different roles in the evaluation process and who has been trained on what, so that each person is aware of who else is on board with the de-labeling protocols and what their roles are.

Perspectives varied in terms of the format they thought the education could be conveyed. Ideas included information dissemination via email, periodic presentations at meetings so people can ask questions, and videos for clinicians and trainees to watch on their own time.

### Training

Participants noted that education to increase knowledge was not enough and that training on how to evaluate patients was necessary to build essential skills and bolster clinicians’ comfort and confidence. Participants also felt it was best to train permanent staff in the de-labeling process, so that they could then champion and train future residents and fellows on the proper protocols.

Most participants emphasized the importance of training for addressing allergic reactions during penicillin allergy challenges. Several mentioned that clinicians may fear patient reactions and thus be hesitant to initiate evaluation. This training could help reduce hesitancy and provide reassurance so that clinicians feel confident and equipped to address any issues that may arise during evaluation.

### Persuasion

Persuasion is achieved by using communication to induce positive or negative feelings or stimulate action. Participants discussed a few communication tactics that might help increase clinician motivation for carrying out evaluations of patients’ penicillin allergies. First, they felt that communicating about the data around benefits of de-labeling might increase positive feelings about evaluation. Information about why de-labeling is important and data on the long-term benefits of de-labeling as opposed to prescribing alternative antibiotics could be included as part of the education strategy discussed above.

Participants also stated how reinforcing that the Allergy specialty service endorses the content of the CDST would give frontline clinicians more confidence in following these algorithms and clinical protocols for managing patients with a penicillin allergy.

### Restriction

Restriction aims to increase the target behavior by creating rules that reduce the opportunity to engage in competing behaviors. For our project, we identified two competing behaviors that clinicians could engage in. One of them is overlooking the opportunities to challenge a patient and remove the allergy label from their health records. The second is prescribing an alternative medicine to treat the infection, which is often perceived as more efficient. In order to restrict these behaviors, leadership should communicate clearly that that de-labeling patients is a priority in the clinic. This could come in the form of top-down directive, or could include additional weight such as creating an official initiative for quality improvement or a local antimicrobial stewardship (AMS) policy mandating de-labeling as a metric to be monitored.

### Environmental restructuring

Another important intervention function included in BCW, environmental restructuring, proposes reshaping the physical or social context in order to achieve a behavior. This function places more emphasis on the external factors that influence behavior change and less on personal agency, which is addressed by the intervention functions including education, training, and persuasion. Our results demonstrated four main areas that could be targeted by environmental restructuring: clarifying roles and responsibilities, rethinking communication systems, addressing staffing demands, and changing the physical environment.

One key area was refining and clarifying the roles and responsibilities of individuals in teams pertaining to penicillin de-labeling. Many participants discussed having a role of a champion who will ‘own’ the process and act as a point person, including developing workflows for tasks, overseeing each team to ensure the completion of challenges, and correctly updating the patient EHR in the system. Having this centralized owner reduces practice variability and “generally works a lot better than asking each individual team or provider to have that on their radar” (PCP 4). The second role recommended by clinicians was a coordinator who will initiate the process by identifying patients who can be evaluated. This person could identify priority patients, such as patients who need penicillin before a surgery or have a higher risk for readmission (e.g. recurrent infections).

Participants also pointed out that restructuring communication systems would enable clearer distribution of responsibilities during the de-labeling process and improve relational connections among interdisciplinary team members. Although increased use of messaging platforms during the pandemic enabled teams to maintain workflows, reliance on virtual and often asynchronous communications also created silos, delays, and misunderstandings about level of importance of particular tasks. Resuming multi-disciplinary rounds and increasing opportunities for in-person huddles were proposed as strategies to address these communication gaps and offer forums for discussing questions about allergy challenge protocols to improve uptake of de-labeling. Rather than discontinuing virtual communications, clinicians suggested protocols to ensure recipients would follow through on recommendations when in-person communication is not possible. Participants also suggested including notes in the EHR system along with a consult order for the next team to facilitate a smoother transition from inpatient to outpatient care or primary care to specialty care. Having alerts to check on patients with a penicillin allergy listed in their EHR but recently prescribed an antibiotic was also proposed.

Hiring additional staff and distributing staff differently was suggested to counteract the competing priorities and lack of bandwidth that were a constant struggle for clinicians in both inpatient and outpatient settings. Staffing shortages exacerbated during the pandemic added to this challenge. Multiple clinicians discussed how help with administrative tasks would enable them to dedicate more time to patient-facing responsibilities. While practices vary among hospitals, some proposed hiring administrative staff to round with teams and handle paperwork to remedy time constraints. Pharmacists also noted that having additional staff to cover weekend shifts would help reach patients who were admitted over the weekend. This would also enable identifying patients with penicillin allergy within 12–24 h after admission, which would help avoid possible delays with hospital discharge due to de-labeling processes. As their bandwidth expands, providers would also have more time to build trust and educate patients who were less willing to agree to getting tested.

Finally, participants suggested that restructuring the physical space so that observation rooms were on the same floor as the clinical team would facilitate nurses and pharmacists to administer the drug challenges. Patients would continue to be monitored by nursing during the process, similar to blood transfusion administration, a protocol which is well established within most hospital systems. This restructuring would allow physicians to continue their normal workflow but allow them to be more easily accessible to nursing if a reaction to the drug challenge occurred.

### Enablement

For our study, we defined enablement as forms of assistance that help clinicians incorporate de-labeling into their everyday workflows. Participants brought up a number of ideas that would enable and enhance uptake of de-labeling. Streamlining and simplifying the EHR system to facilitate easy incorporation of tasks into workflows was proposed as a critical modification, including standardizing de-labeling order sets across the inpatient and outpatient settings and adding reminders. Outpatient pharmacists indicated willingness to take on more de-labeling tasks if they were provided with scripts and/or a one-page information sheet that they could refer to when talking to patients.

## Discussion

The high degree of mislabeling of penicillin allergy coupled with the prevalence of patients with reported penicillin allergy has created a need to de-label a large number of patients. Past literature have described clinical decision rules and protocols for conducting direct oral challenges in low risk penicillin allergy [18–22]. In addition, there is increasing awareness of the need to further study how to best apply validated protocols with a lens on sustainability and scalability [23–27]. Using an implementation science approach can enhance processes to best design and scale interventions to expand access to penicillin allergy evaluation through guideline-based protocols.

**Fig. 4:**
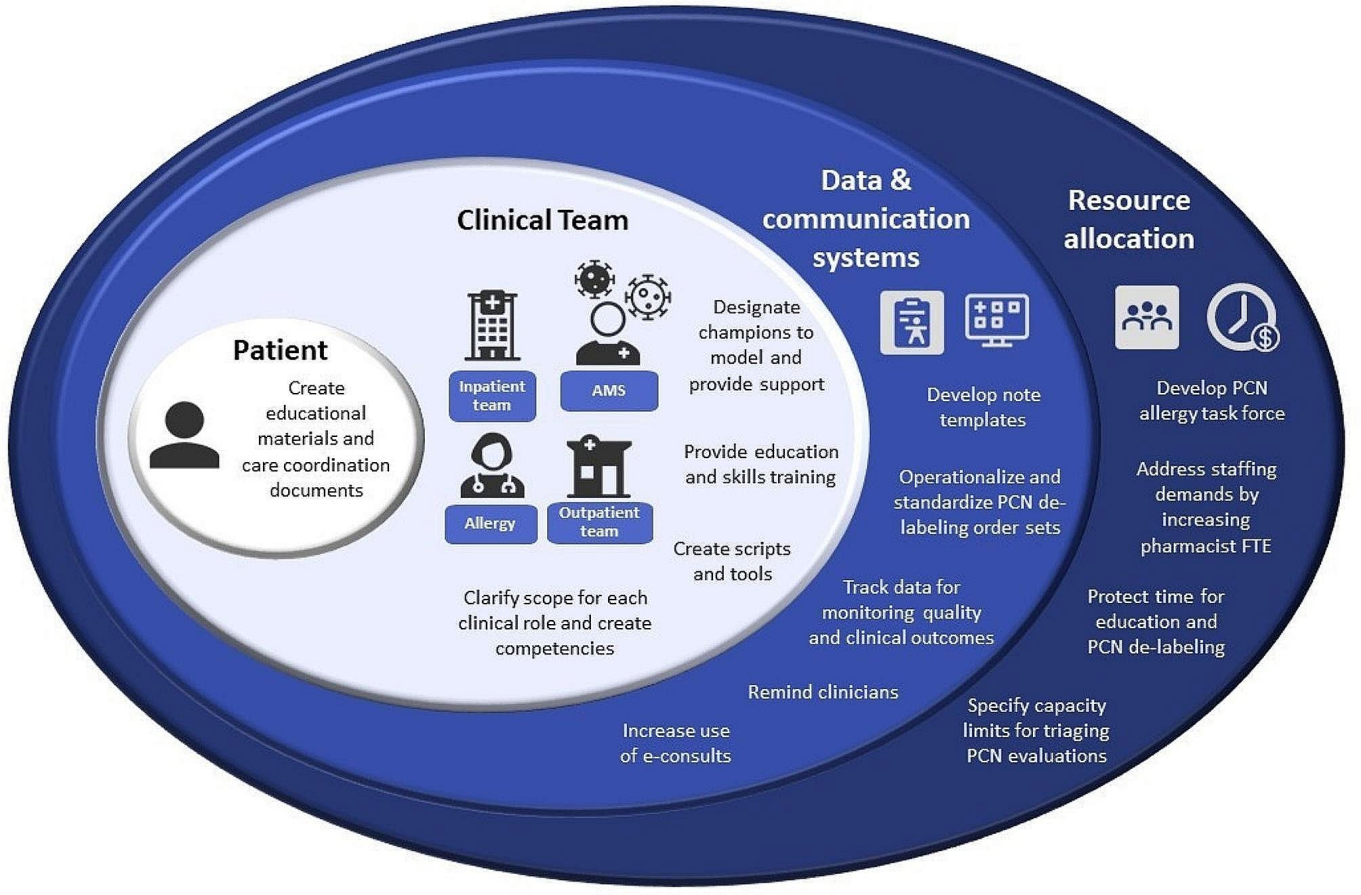
Components of a multi-level multi-modal intervention to increase penicillin allergy de-labeling. AMS: Anti-microbial stewardship; PCN: Penicillin; FTE: Full-time equivalent

Our prior study demonstrated that better role clarification, opportunities to develop necessary skills, and dedicated resources are needed to overcome barriers to implementing de-labeling interventions. Our current study describes a process to strategically leverage these findings to develop evidence-based interventions to overcome these barriers. The steps we follow to this end are twofold: (1) we apply the process of IM to a clinical gap in allergy care (2) we identify theory-driven, actionable strategies to improve penicillin allergy evaluation processes. IM has been shown to be effective in changing chronic disease management and screening in other conditions [28]. We will utilize this process to develop a multi-level multi-modal intervention (Fig. 4). Using this approach will translate Allergy and Immunology evidence-based practices into clinical applications and close the clinical gaps of care in drug allergy and other allergic diseases.

Current penicillin allergy evaluation recommendations have advocated for point of service de-labeling of patients, particularly if they are low risk, as it allows efficient tailoring of antimicrobial prescribing [29]. Point of service de-labeling empowers teams to address mislabeled allergies at the patient encounter rather than referring them to outpatient care. Providing clinicians with necessary training and resources such as toolkits and guidelines that they could refer to if needed is an important first step in improving implementation and will need to be followed by models and processes that address modifying environments and human behavior to improve application of currently available toolkits [30]. Our study discussed a process method of IM to determine actionable steps to apply and study best practices on how to apply and tailor these tools for local needs. These tools, along with a comprehensive understanding of the system-levels barriers that influence point of demand decision making, will facilitate reach and scalability of penicillin allergy de-labeling interventions. We specifically describe the need to address intervention functions in the areas of environmental restructuring and enablement.

Our results have shown that to implement point of service penicillin allergy de-labeling practices, a multilayered approach is needed that incorporates education to address knowledge barriers, training to develop skills to identify low-risk patients and treat possible allergic reactions, and clarification on how roles are distributed. Additionally, developing a system of champions, improving communication systems, and restructuring the environment around the healthcare team are demonstrated as critical components of an effective behavior change intervention. Our results demonstrate similarities with previous literature. Fahim et al. [31] utilized a similar process to select interventions to improve the multidisciplinary cancer conferences decision making processes. Similarly, Gallant et al. [32] presented that a multi-levelled intervention was needed to overcome barriers to vaccine uptake.

Our study is unique in that it describes a scientific process of IM to demonstrate how a multi-method study approach can establish a process and a package of implementation strategies to drive behavior and system change. By targeting the psychological underpinnings of current behavioral and environmental barriers to penicillin de-labeling, we developed a transparent process that demonstrates which intervention components are more likely to yield uptake and sustainment. Limitations of our study involve single site data collection and the impact of pandemic-level staffing and pressures for our inpatient clinicians. We also will need to study the applications of our designed interventions in a repetitive process improvement approach to describe efficacy, scalability and sustainability of our implementation package. Future work can be directed on a mixed methods approach focused on efficacy and implementation outcomes of our proposed implementation strategies. Additionally, prior literature in antimicrobial stewardship have indicated that establishing a metric, linked to strategic institutional outcomes and prescribing practices may bolster antimicrobial stewardship institutional initiatives [33–35]. Similarly, the development of data reporting tools or dashboards related to numbers of patients de-labeled from penicillin allergy and its relevance to antibiotic prescribing practices may be important future areas of research.

### Conclusion and future directions

Given the direct impact penicillin allergy has on antimicrobial stewardship and patient safety, there is a need to support multilevel and multidisciplinary approaches to safely assess penicillin allergies. Key to the success of these interventions is a careful study of process and contextual factors influencing penicillin allergy evaluation and de-labeling. Successful programs and penicillin allergy toolkits have been launched to increase knowledge dissemination and provide procedural tools to promote penicillin allergy evaluation. However, the results of our study show that a comprehensive study of barriers and contextual factors can identify system-level issues that may impede the reach and sustainable use of current state interventions. The IM approach provides a framework to further develop implementation packages to study how to best apply evidence-based penicillin allergy interventions to allow scalability, sustainability and improve efficacy of our interventions.

### Electronic supplementary material

Below is the link to the electronic supplementary material.

Supplementary Material 1

## Data Availability

The datasets analyzed during the current study are available from the corresponding author upon reasonable request.

## Abbreviations

AMS: Antimicrobial Stewardship
BCW: Behavior Change Wheel
CDST: Clinical Decision Support Tool
CME: Continuing Medical Education
COM-B: Capability, Opportunity, and Motivation- Behavior
EHR: Electronic Health Record
ERIC: Expert Recommendations for Implementing Change
F2F: Face to Face
FTE: Full time Equivalent
IM: Implementation Mapping
N/A: not applicable
PCN: Penicillin
PCP: Primary Care Provider
TDF: Theoretical Domains Framework
