# Supplementary material for: Leveraging implementation science theories to develop and expand the use of a penicillin allergy de-labeling intervention
Source: BMC Health Serv Res. 2024 Aug 26;24:987. doi: 10.1186/s12913-024-11364-7 (PMC11348780; doi:10.1186/s12913-024-11364-7)
Supplement: Supplementary file 1 — Supplementary Material 1 [file 12913_2024_11364_MOESM1_ESM.docx]

**Provider Interview Questions**

**First we would like to understand your perceptions of allergy delabeling processes and roles here at the VA.**

- How do you think allergy de-labeling will improve patient outcomes? Relevant patient outcomes would be: more judicious antibiotic use and better treated infections, decreased healthcare costs and possibly decreased admissions to the hospital
  - Are there specific patients who you think would benefit from delabeling more than others?
- How often do you evaluate patients with penicillin allergy?
  - What has been your experience with these evaluations?
- Do you see a role for primary care in penicillin allergy evaluation and delabeling? If so, what role?
  - Are there any members on the PACT team who may help with this? Which members?
    - How do you see the pharmacist helping?
  - Are you aware of any processes at the VA regarding pcn allergy delabeling?

**Next we’d like to discuss specific aspects of drug allergy evaluation.**

- What are the key questions to ask when taking a history for patients with penicillin allergy?
- Which parts of clinical history do you see as the biggest predictors of risk of future reaction?
- Once you take the clinical history, do you know the next steps of evaluating a patient with penicillin allergy?
- How comfortable do you feel in initiating a conversation with your patients who are good candidates for de-labeling?
  - Would you want example scripts for talking to patients?
  - Do you have patients who ask about getting tested, and do you feel comfortable talking to them about it?
- What other resources would you need to take a history or do a test dose challenge?

**Now we’d like to discuss the barriers to administering penicillin de-labeling.**

- What do you think is the most significant barrier to incorporating de-labeling into your work?
- What other significant barriers are there to incorporating de-labeling into your work?
- Time constraints
  - What is preventing you from spending time on delabeling?
  - How do you feel about evaluating patients for penicillin allergy if it is not related to their visit?
  - Are there particular groups of patients for whom you would be less likely to initiate de-labeling? For example, particular demographic groups?
  - For which patients would you be *more* likely to initiate de-labeling?
- Reservations about allergy challenges (re: managing adverse reactions)
  - Are there patients you think will have adverse outcomes from penicillin drug challenges?
    - If so, how does this influence your decision to initiate the process?
  - How comfortable are you with treating patients who may have adverse reactions during the drug challenge?
  - Do you feel at risk for discipline, peer review, other negative consequence if an unanticipated side effect happens on a patient you assessed and attempted to de-label from penicillin allergy?
- Thinking about the barriers we have discussed, what suggestions do you have to overcome these barriers?
  - If time ask about how they would overcome specific barriers
  - What other resources would you need (if you were) to become comfortable de-labelling penicillin allergies in a patient record?

**Considering all we’ve discussed, we are interested in hearing your ideas for process improvement.**

- Do you have suggestions or ideas on how to implement this in primary care?
- If you were to get more involved with allergy evaluation and delabeling, what would be the ideal workflow?
- What would motivate you and other physicians to recommend/administer delabeling?
  - Are there times you feel it would benefit you and the patient to do a test dose challenge?
  - Probe on whether incentives would change their decision
  - What skills would you need to de-label penicillin allergies in a patient record?
- What thought processes might guide your decision to initiate/administer penicillin de-labeling in your clinic?
